# Identifying candidate drivers of alcohol dependence-induced excessive drinking by assembly and interrogation of brain-specific regulatory networks

**DOI:** 10.1186/s13059-015-0593-5

**Published:** 2015-02-02

**Authors:** Vez Repunte-Canonigo, William Shin, Leandro F Vendruscolo, Celine Lefebvre, Lena van der Stap, Tomoya Kawamura, Joel E Schlosburg, Mariano Alvarez, George F Koob, Andrea Califano, Pietro Paolo Sanna

**Affiliations:** Molecular and Integrative Neuroscience Department, The Scripps Research Institute, La Jolla, CA USA; Committee for the Neurobiology of Addictive Disorders, The Scripps Research Institute, La Jolla, CA USA; Department of Biological Sciences, Columbia University, New York, NY 10027 USA; Department of Systems Biology, Herbert Irving Comprehensive Cancer Center, Columbia University, New York, NY 10032 USA; Department of Biomedical Informatics, Herbert Irving Comprehensive Cancer Center, Columbia University, New York, NY 10032 USA; Institute for Cancer Genetics, Herbert Irving Comprehensive Cancer Center, Columbia University, New York, NY 10032 USA; Department of Biochemistry and Molecular Biophysics, Hammer Health Sciences Center, Columbia University, New York, NY 10032 USA; Cancer Regulatory Network Program, Herbert Irving Comprehensive Cancer Center, Columbia University, New York, NY 10032 USA; Current affiliation: Inserm Unit U981, Gustave Roussy Institute, Villejuif, France; Current affiliation: Intramural Research Program, NIDA-NIH, Baltimore, MD 21224 USA; Current affiliation: National Institute on Alcohol Abuse and Alcoholism, Rockville, MD 20852 USA; The Scripps Research Institute, 10550 North Torrey Pines Road, La Jolla, CA 92037 USA

## Abstract

**Background:**

A systems biology approach based on the assembly and interrogation of gene regulatory networks, or interactomes, was used to study neuroadaptation processes associated with the transition to alcohol dependence at the molecular level.

**Results:**

Using a rat model of dependent and non-dependent alcohol self-administration, we reverse engineered a global transcriptional regulatory network during protracted abstinence, a period when relapse rates are highest. We then interrogated the network to identify master regulator genes that mechanistically regulate brain region-specific signatures associated with dependent and non-dependent alcohol self-administration. Among these, the gene coding for the glucocorticoid receptor was independently identified as a master regulator in multiple brain regions, including the medial prefrontal cortex, nucleus accumbens, central nucleus of the amygdala, and ventral tegmental area, consistent with the view that brain reward and stress systems are dysregulated during protracted abstinence. Administration of the glucocorticoid antagonist mifepristone in either the nucleus accumbens or ventral tegmental area selectively decreased dependent, excessive, alcohol self-administration in rats but had no effect on non-dependent, moderate, alcohol self-administration.

**Conclusions:**

Our study suggests that assembly and analysis of regulatory networks is an effective strategy for the identification of key regulators of long-term neuroplastic changes within specific brain regions that play a functional role in alcohol dependence. More specifically, our results support a key role for regulatory networks downstream of the glucocorticoid receptor in excessive alcohol drinking during protracted alcohol abstinence.

**Electronic supplementary material:**

The online version of this article (doi:10.1186/s13059-015-0593-5) contains supplementary material, which is available to authorized users.

## Background

Development of alcoholism involves a complex interplay of distinct molecular mechanisms. Thus, elucidating the key molecular determinants of the transition to dependence will require innovative genome-wide modeling strategies [1]. In this study, we tested whether a systems biology approach that has been highly effective in elucidating drivers of cancer [2-7] and developmental phenotypes [8,9] could be effective in elucidating the mechanisms that control the neuroadaptive changes associated with excessive drinking. This approach can potentially lead to novel insights into the disease process and ultimately more effective therapeutic targets [1].

In order to produce the first genome-wide, transcriptional model, or interactome, of a mammalian central nervous system (CNS), we used the Algorithm for the Reconstruction of Accurate Cellular Networks (ARACNe) [10], which is based on an information-theoretic approach for the inference of the targets of transcription factors (TF), that is, the TF-regulon. Specifically, we analyzed a collection of 96 gene expression profiles (GSE60966) from microdissected brain regions of the central reward and stress pathways in a rat behavioral model of dependent and non-dependent alcohol self-administering rats [11,12]. In this model, rats are chronically exposed to intermittent alcohol vapor to intoxication to induce dependence. Dependent rats in this model rapidly escalate their alcohol intake during repeated withdrawal periods and show compulsive responding for alcohol [11-13]. In particular, dependent rats self-administer sufficient amounts of alcohol to reach blood alcohol levels comparable to excessive drinking in alcohol use disorders humans [14,15], and manifest physical and motivational (anxiety, dysphoria, and hypohedonia) signs of withdrawal during acute and protracted abstinence periods [11-14,16,17]. Compulsive alcohol seeking in dependent rats is reflected by increased progressive-ratio responding and persistent alcohol consumption despite punishment [13]. Dependent rats also exhibit electrophysiological changes in the extended amygdala during withdrawal [16,18].

We interrogated the CNS transcriptional-interactome with the Master Regulator Inference Algorithm (MARINa) [2,9] to identify the key genes that drive the expression of the specific gene signatures associated with alcohol dependence (master regulator genes (MRs)). Rather than selecting candidate genes based on existing knowledge, for example, scientific literature, or on differential expression, MARINa computes the enrichment in differentially expressed genes of the ARACNe-inferred targets of a TF (regulon). This is used to assess the TF’s role in implementing the gene expression signature representative of the phenotypes of interest (for example, alcohol non-dependent vs. alcohol dependent, in the present setting) [2,9]. This is motivated by the fact that many TFs are post-translationally regulated and, thus, their transcriptional activity may not be directly proportional to their expression levels. This methodology has been highly successful in elucidating key biological drivers of cancer and developmental phenotypes [2,7-9,19,20], where MARINa-inferred MRs have been validated as causally related to phenotype presentation with high probability (>70%). However, this approach had not been previously tested in the context of complex CNS diseases.

Here, we report on several transcription factors predicted by MARINa as key drivers of brain-region-specific gene expression signatures associated with a history of alcohol dependence in rats. Of these, Nr3c1 (Table 1), the gene coding for the glucocorticoid receptor (GR), was one of the highest-ranking MRs in several brain regions from which signatures were derived, including the central nucleus of the amygdala (CeA), medial prefrontal cortex (mPFC), and core sub-region of the nucleus accumbens (NAc), where it was recruited both in the context of non-dependent and dependent alcohol drinking; and the shell of the NAc and the ventral tegmental area (VTA), where Nr3c1/GR was recruited selectively by a history of alcohol dependence. These results suggest that glucocorticoid-dependent neuroadaptive changes in these brain regions may contribute to excessive drinking during protracted abstinence. We have recently observed that systemic GR antagonism with mifepristone (RU38486) blocks compulsive alcohol drinking during protracted abstinence [13]. In the present study, we tested the functional role of the GR in specific brain regions such as the NAc and VTA, where Nr3c1/GR was unexpectedly predicted to be a high-ranking master regulator, via intracerebral (IC) administration of mifepristone. Here we found that GR antagonism with mifepristone in either the NAc or the VTA selectively decreased escalated alcohol intake in rats with a history of alcohol dependence.

**Table 1:** **List of gene names and symbols mentioned in the manuscript**

**Symbol**	**Gene name**
Cdh2	Cadherin 2
Cdk5	Cyclin-dependent kinase 5
Hopx	HOP homeobox
Hsp90aa1	Heat shock protein 90 kDa alpha (cytosolic), class A member 1
Mapk3	Mitogen-activated protein kinase 3 (ERK1)
Med1	Mediator complex subunit 1
Med14	Mediator complex subunit 14
Ncoa3	Nuclear receptor coactivator 3
Ncor2	Nuclear receptor corepressor 2
Nfkb1	Nuclear factor of kappa light polypeptide gene enhancer in B-cells 1
Nr3c1	Nuclear receptor subfamily 3, group C, member 1 (glucocorticoid receptor)
Nr3c2	Nuclear receptor subfamily 3, group C, member 2 (mineralocorticoid receptor)
Pou2f1	POU domain, class 2, transcription factor 1
Psip1	PC4 and SFRS1 interacting protein 1
Smarca4	SWI/SNF-related, matrix-associated, actin-dependent regulator of chromatin, subfamily a, member 4
Smarcad1	SWI/SNF-related, matrix-associated, actin-dependent regulator of chromatin, subfamily a, containing DEAD/H box 1
Trim28	Tripartite motif-containing 28
Txn1	Thioredoxin 1
Ywhah	Tyrosine 3-monooxygenase/tryptophan 5-monooxygenase activation protein, eta polypeptide

These results identify a key role of regulatory networks downstream of GR in the neuroadaptive changes that take place in the progression from alcohol naive to alcohol drinking and from non-dependent alcohol drinking to alcohol dependence, and support the potential of the present systems biology approach to deconvolve dysregulated gene regulatory networks in diseases affecting the CNS and to identify therapeutic targets for excessive alcohol drinking.

## Results

### Alcohol self-administration and induction of dependence

**Figure 1:**
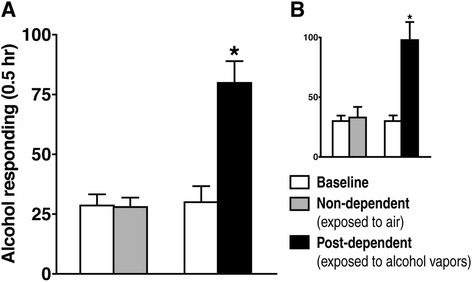
Escalation of alcohol intake induced by chronic, intermittent alcohol vapor exposure in rats. **(A)** Responding for alcohol by the animals used for the microarray dataset in the gene network analysis. A total of 96 gene expression profiles from eight brain regions (mPFC, BLA, dorsolateral and ventrolateral BNST, CeA, core and shell of NAc, VTA) from four rats per group in this experiment were used to reconstruct the transcriptional regulatory network, or transcriptional-interactome. White bars show the average operant responding for ethanol over the last 11 self-administration sessions prior to induction of dependence (Baseline) for both the rats exposed to alcohol vapor to induce dependence and non-dependent rats exposed to air in the same apparatus for control. Operant responding during acute withdrawal after 4-week exposure to chronic, intermittent alcohol vapor (or air for control) is shown by the right hand bars: black bar = dependent rats and grey bar = non-dependent rats. The average number of presses for ethanol in the dependent group during acute withdrawal was significantly increased over the non-dependent group and over the baseline response level of the same rats; conversely, in animals exposed to air instead of alcohol vapors for control, the average number of presses was unchanged from their baseline. *P <0.01 from the other three groups. **(B)** Responding from all the rats in the study (n = 10) including the four per group used for the microarray profiling and the others that were used for RT-PCR validation (Y-axis scale as in A); *P <0.01 from the other three groups. Rats were sacrificed during protracted abstinence (3 weeks after the last self-administration session and vapor exposure).

Rats were trained to orally self-administer alcohol in a concurrent, two-lever, free-choice contingency procedure as previously reported [13]. Following acquisition of self-administration, rats were allowed to self-administer unsweetened alcohol (10%; w/v) for 4 weeks and were then assigned to two groups matched by levels of responding: one group (dependent group) was exposed to chronic, intermittent (14 h ON/10 h OFF) ethanol vapors (blood alcohol levels between 175 mg% and 225 mg%) for 4 weeks to induce dependence; the other group (non-dependent group) was not exposed to ethanol vapor. After 1 month of vapor exposure, rats were again tested during acute withdrawal (6 to 8 h after removal from the vapor chambers). As expected, alcohol vapor-exposed rats self-administered significantly greater amounts of alcohol than control rats not exposed to alcohol vapor during acute withdrawal (Figure 1). Rats were sacrificed during protracted abstinence (3 weeks after the end of alcohol vapor exposure) along with age-matched alcohol naive rats.

### Regulatory network assembly

We reconstructed a transcriptional-interactome from a dataset of 96 gene expression profiles (GEP) from eight brain regions believed to be relevant in alcohol’s reinforcing properties using the Affymetrix RN230.2 platform. Specifically, the following brain regions were microdissected and analyzed from non-dependent and dependent alcohol self-administering rats as well as age-matched alcohol naive rats: (a) mPFC, (b) shell and (c) core NAc sub-regions, (d) CeA), (e) BLA, (f) dorsolateral and (g) ventral bed nucleus of the stria terminalis (BNST), and (h) VTA (Additional file 1: Figure S1). To improve the accuracy and reproducibility of the analyses, the Cleaner algorithm [21] was used to normalize the GEP data. Cleaner maps individual Affymetrix probes to the most recent Refseq transcript database, thus eliminating probes mapping to multiple genes or to incorrect and/or intragenic regions [21]. Cleaner also clusters probes into probesets, based on probe correlation across the entire GEP dataset, producing probesets that optimally monitor the expression of individual alternative gene transcripts, thus excluding low-quality and incorrectly matched probes [21]. Cleaner identified 7,019 high-quality probesets in the Affymetrix RNU230.2 GEP data, representing the transcripts of 6,818 expressed genes, down from the 19,456 probesets originally represented on the microarray platform. Thus, only about one-third of the monitored probes were considered to be high quality and mapped to expressed genes.

The Cleaner-normalized dataset was then processed by ARACNe to produce a genome-wide transcriptional-interactome. ARACNe first computes the Mutual Information (MI), *I*(*TF*; *t*), between each transcription factor, *TF*, and candidate target, *t*, in the dataset [10]. TF-target pairs are considered as candidate interactions if their MI is statistically significant (*P* ≤0.05, Bonferroni corrected for multiple hypothesis testing). However, indirect interactions (that is, via any other TF) are removed, based on the Data Processing Inequality property of information theory [22]. The ARACNe-inferred transcriptional network included 78,090 predicted TF-target interactions between 664 TFs and 6,716 targets, with an average of about 100 targets per TF (see methods for details on the ARACNe parameters used for this analysis).

### Regulatory network interrogation and validation

**Figure 2:**
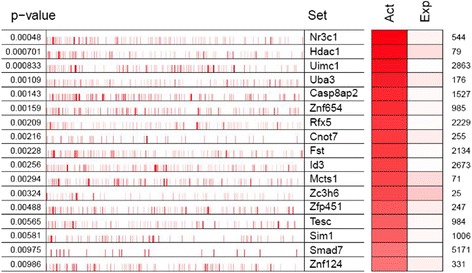
MARINa results showing candidate MRs driving the alcohol non-dependence vs. dependence differentially expressed gene signature in the core sub-region of the nucleus accumbens (NAc) sorted by their differential activity in alcohol dependence. Each row of the plot shows the result of MARINa for each MR candidate. The *P* value associated with each MR candidate is shown on the left. The MARINa-predicted differential activity (Act), differential expression (Exp) and are shown on the right in shades of red: higher levels of Exp or Act are indicated in dark red, while lower levels are indicated in light red or pink. The rank of Exp of each MR candidate is also shown on the right side of the plot. The red lines in the middle of the plot indicate ARACNe predicted targets of each MR candidate. The position of each line on the horizontal axis corresponds to its rank in the gene list, which is determined by its differential expression. More differentially expressed genes being shown toward the left, and the less differentially expressed genes displayed toward the right. The identity and differential expression of the ARACNe-predicted targets of each MR candidate can be found in Additional file 3 in the online version of this manuscript.

The CNS transcriptional-interactome was then interrogated using the MARINa algorithm [2,9] to identify candidate MRs driving the gene expression signatures associated with non-dependent and dependent alcohol self-administration. This includes all genes, ranked according to their differential expression in rats with a history of dependent vs. non-dependent self-administration, as well as in both groups compared with alcohol naive rats. MARINa analyzes each TF in the transcriptional-interactome by measuring the enrichment of its ARACNe-inferred targets in the gene expression signature. The statistical significance of the enrichment is computed by Gene Set Enrichment Analysis (GSEA) [23]. Figure 2 shows the results of MARINa for the core sub-region of the nucleus accumbens (NAc) sorted by the TFs or MR candidates’ differential activity (NES) in alcohol dependence. Each row of the plot shows the result of MARINa for statistically-significant TFs or MR candidates. The number of MRs predicted in each region, as well as the top-25 MR candidates for each region and comparison are shown in Additional file 1: Table S1 and Figures S4 and S5. The overlap among MRs inferred from different brain regions and the associated *P* value based on Fisher’s exact test (FET) can be found in Additional file 1: Table S2. The identity and differential expression of the ARACNe-predicted targets of each MR candidate can be found in Additional files 2 and 3 in the online version of this manuscript.

Of particular interest, the core and shell sub-regions of the NAc, as well as the VTA, showed a highly significant fraction of overlapping MRs (*P* <0.01), suggesting that some common neuroadaptations may affect elements of the reward-motivational systems (Additional file 1: Tables S2a and b). The VTA also showed significant MR overlap with elements of the extended amygdala: the CeA (*P* <0.01), and dorsolateral BNST (*P* <0.01) in the non-dependent vs. dependent gene signature (Additional file 1: Table S2b). While regions with the most significant MR overlap were generally closer in the GEP hierarchical clustering analysis (Additional file 1: Figure S2 and Table S2a), this was not always the case. For instance, the dorsolateral BNST and ventral BNST shared only one common MRs despite similar gene expression programs (Additional file 1: Figure S3 and Table S2b). We found no candidate MRs that were enriched in every region, suggesting that the effect of alcohol may be largely mediated by region-specific neuroadaptations (Additional file 1: Figure S3 and Table S2b). We also found that for the non-dependent vs. dependent gene signatures, the most significant MR candidates were in the NAc core, shell, VTA, CeA, BLA and dorsal BNST regions, whereas the most significant MR candidates in the alcohol naive vs. non-dependent signature were in the mPFC, NAc core, NAc shell, dorsal and ventral BNST, and VTA regions (Additional file 1: Table S1). These results suggest a differential and progressive recruitment of brain regions of the reward and stress system in the transition to dependence.

**Figure 3:**
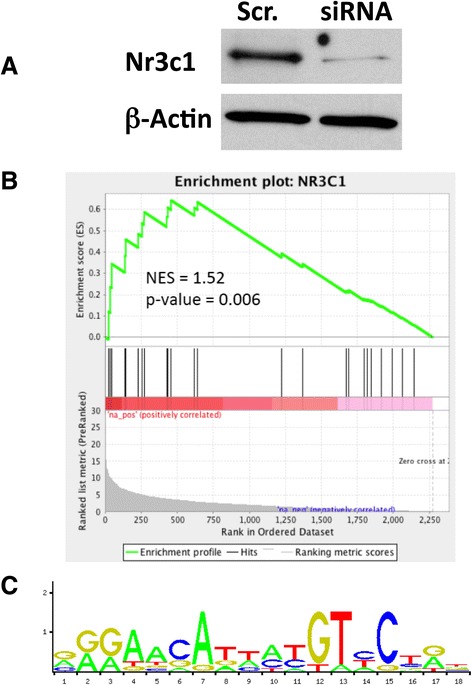
Validation of ARACNe-predicted Nr3c1/GR regulon by siRNA-mediated downregulation of Nr3c1/GR followed by microarray analysis. **(A)** Nr3c1/GR was downregulated by use of a synthetic siRNA (Applied Biosystems) in the rat striatal medium spiny neuron cell line M213 relative to an aspecific ‘scrambled’ siRNA (Scr.) control. **(B)** We performed microarray profiling and used gene expression differences between cells treated with siRNA to Nr3c1/GR and the ‘scrambled’ siRNA control to interrogate the Nr3c1 regulon (that is, targets of Nr3c1/GR predicted by ARACNe) with the Gene Set Enrichment Analysis (GSEA) [23]; n = 4. Results showed enrichment of the genes in the Nr3c1/GR regulon among the genes whose expression is affected by siRNA-mediated downregulation of Nr3c1/GR in M213 cells (GSEA result: NES = 1.28, *P* <0.004). **(C)** Sequence logo of Nr3c1/GR binding site from JASPAR database [24].

**Figure 4:**
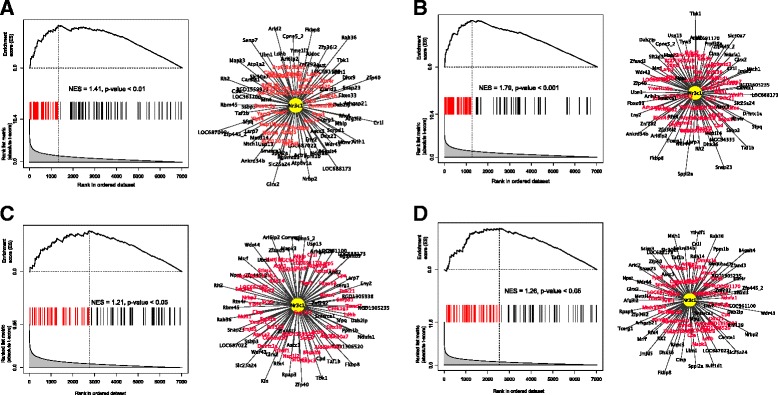
Targets of Nr3c1/GR as predicted by the ARACNe-generated transcriptional-interactome. Targets of Nr3c1/GR in the NAc core **(A)**, shell **(B)**, and VTA **(C)** differentially regulated in the non-dependent vs. dependent signature. Targets of Nr3c1/GR in the CeA **(D)** in the naive vs. non-dependent signature. For each comparison, the results of GSEA for Nr3c1/GR targets in the corresponding signature are shown on the left and the Nr3c1/GR regulon on the right. In the right panel, the distance between Nr3c1/GR and each target represents the degree of differential expression in the signature. More differentially expressed genes are shown closer to Nr3c1/GR, and less differentially expressed genes are shown further away. Genes in the leading edge of the GSEA analysis are shown in red. These are ones that contribute most significantly to the enrichment of Nr3c1/GR, and are the most strongly regulated targets of Nr3c1/GR. The GSEA panel on the left shows the enrichment plot, NES and associated *P* value. As with the regulon plot, the genes in the leading edge are also shown in red.

Among the MRs inferred in the analysis, Nr3c1, the gene coding for the GR, was one of the highest ranking on the basis of differential activity (for example, Figure 2). We thus assessed the functional relevance of the network predictions through behavioral validation of this gene in the specific brain regions identified by the analysis. As shown in Figure 3, we first validated the accuracy of the ARACNe-inferred Nr3c1/GR regulon (that is, the set of its inferred transcriptional targets; the complete ARACNe-inferred Nr3C1/GR regulon is shown in Figure 4, and the identity and differential expression of the ARACNe-predicted targets of Nr3c1 in both the alcohol naive vs. non-dependent, and non-dependent vs. dependent signatures can be found in Additional files 4 and 5 in the online version of this manuscript). ARACNe-inferred targets of the Nr3c1 TF (that is, the Nr3c1 regulon) were found to be significantly enriched in genes differentially expressed following shRNA-mediated silencing of this TF (*P* <0.004 by GSEA analysis [23], Figure 3A and B). Promoters of ARACNe-inferred Nr3c1/GR targets were found to be significantly enriched in canonical Nr3c1/GR binding sites (Figure 3C; *P* <0.05), compared to randomly selected promoters from non-ARACNe predicted targets. Binding site enrichment analysis was performed using the position-specific-scoring-matrix for Nr3c1/GR (MA0013.1) downloaded from the JASPAR database [24]. The position-specific-scoring-matrix was scored using the maximum-likelihood method outlined by Conlon *et al.* [25]. Taken together, these independent analyses suggest that the ARACNe-inferred regulon is highly enriched in bona-fide, physical Nr3c1/GR targets. Note that since ARACNe does not use any sequence related knowledge, such as the presence of binding sites in the promoters of predicted targets, the evidence from the two analyses is statistically independent and can thus be combined.

**Figure 5:**
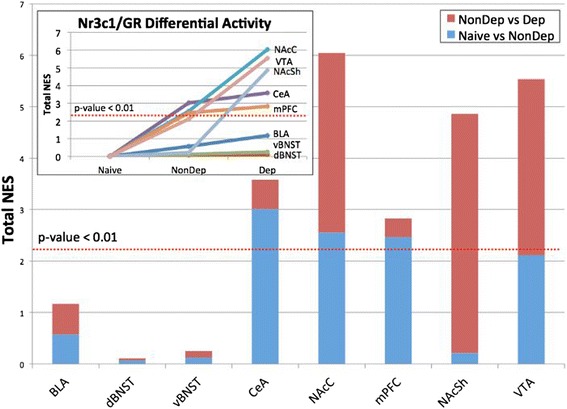
Results of MARINa analyses for Nr3c1/GR in alcohol naive, non-dependent and dependent rats. Both the main stacked-column graph and line graph inset show the total differential activity of Nr3c1/GR across eight regions in the control (alcohol naive), non-dependent, and dependent groups. Differential activity is measured by the normalized enrichment score (NES) in each region, calculated as the differential activity going from alcohol naive to non-dependent and from non-dependent to dependent. Nr3c1/GR is activated in non-dependent alcohol self-administering rats and increases to a lesser extent after the transition to dependence. In regions like the VTA and NAc core, Nr3c1/GR activity increases progressively from alcohol naive to non-dependent to dependent rats, while in the NAc shell, significant Nr3c1/GR activation is seen only after the transition to dependence. The dashed red line in both plots indicate significantly increased differential activity, which corresponds to a total NES of approximately 2.33 and *P* <0.01.

MARINa analyses of alcohol naive vs. non-dependent and dependent alcohol self-administering rats revealed that Nr3c1/GR is recruited in a brain-specific manner during the progression from alcohol naive to non-dependent and, in turn, to dependence. Nr3c1/GR is activated in the CeA and mPFC in non-dependent alcohol self-administering rats, as compared to alcohol naive rats, and it is further increased only slightly in these regions after the transition to dependence. In contrast, Nr3c1/GR activity increases progressively during alcohol exposure and dependence in the VTA and NAc core, while in the NAc shell, Nr3c1/GR activation is seen only after the transition to dependence (Figure 5 and Additional file 1: Table S3a and b).

**Figure 6:**
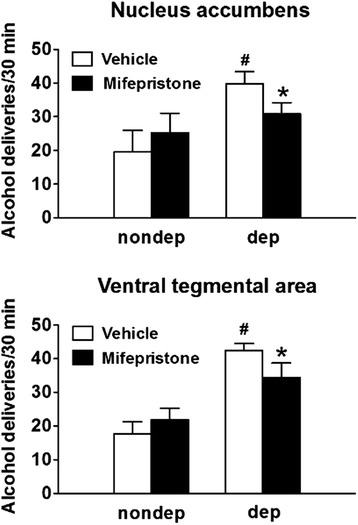
Administration of the GR antagonist mifepristone (RU38486) in either the NAc or VTA selectively decreased alcohol self-administration in rats with a history of alcohol dependence (dep) during protracted abstinence. The rats were made dependent on alcohol by chronic, intermittent vapor exposure and then removed from the vapor chambers. Non-dependent rats were not exposed to alcohol vapor (nondep). The rats were prepared with guide cannulae for bilateral injections of mifepristone (30 μg/0.3 μL/side) or vehicle into the nucleus accumbens and ventral tegmental area. The rats were tested for alcohol self-administration during protracted abstinence (1 to 2 months post-vapor) 90 min after mifepristone administration. The data represent mean and standard error. ^#^ *P* <0.05, significant difference from vehicle-treated non-dependent rats. *P <0.05, significant difference from vehicle-treated dependent rats. n = 7 per group.

We then investigated the functional role of the GR on alcohol self-administration, through GR pharmacological antagonism in specific brain regions in which Nr3c1/GR was found to be a high-ranking master regulator, including the NAc and VTA. Rats were trained to self-administer alcohol and exposed to alcohol vapor to induce dependence (or air, for the purpose of control). The rats were implanted bilaterally with guide cannulae aimed at the VTA and the NAc, including both core and shell subregions. The effects of intracerebral microinjection of mifepristone (30 μg/0.3 μL/side) were investigated during protracted abstinence (Figure 6). As expected, vehicle-treated rats with a history of alcohol dependence displayed escalated alcohol intake during protracted abstinence compared with vehicle-treated non-dependent rats (*P* <0.05). Mifepristone injected into the NAc significantly reduced alcohol self-administration in dependent rats without altering alcohol intake in non-dependent rats (group vs. treatment interaction: F_(1, 12)_ = 9.0, *P* <0.05; Fisher’s LSD post hoc test: *P* <0.05). Similar results were obtained with mifepristone injection into the VTA: vehicle-treated dependent rats displayed higher alcohol intake compared with vehicle-treated non-dependent rats (*P* <0.001), and GR blockade with mifepristone significantly reduced alcohol intake in dependent rats (F_(1, 12)_ = 6.9, *P* < 0.05). Water intake was not affected by group or treatment (data not shown).

We then sought to validate a second MR of alcohol dependence. We selected the transcription coactivator Psip1, which was found to be differentially activated by alcohol dependence in the CeA and was not, to our knowledge, previously implicated in the motivation for alcohol. Viral vector-mediated Psip1 over-expression in the CeA significantly decreased compulsive-like alcohol drinking in rats with a history of alcohol dependence (*P* <0.05) but not in non-dependent rats (Additional file 1: Figure S6). Thus, Psip1 is a novel gene with a functional role in excessive alcohol drinking in the setting of alcohol dependence.

Lastly, we computed intra-network functional similarity to generate new hypotheses on candidate GR transcription co-factors (Tfco) and signaling molecules associated with protracted abstinence in dependent rats by using the CNS transcriptional-interactome and the CNS Signaling Molecule interactome, which was constructed using the same parameters as the CNS transcriptional-interactome and a list of known signaling molecules regulating the GR instead of TFs [5,22]. Correlation was calculated between each TF or signaling molecule with more than 25 ARACNe-inferred targets. Target correlation was calculated using Pearson correlation, and statistical significance was calculated by estimating the correlations between random regulons of the same size. Eight signaling molecules (Cdk5, Ywhah, Mapk3, Cdh2, Hsp90aa1, Med14, Txn1, Med1) had significant correlation with Nr3c1/GR activity, as shown by the heatmap in Additional file 1: Figure S7. Additionally, Nr3c1/GR activity was significantly correlated with nine TfcoTFs (Ncoa3, Ncor2, Smarca4, Trim28, Nfkb1, Hopx, Smarcad1, Nr3c2, Pou2f1), as shown in Additional file 1: Figure S8.

## Discussion

Alcoholism is a chronically relapsing disorder characterized by a compulsion to seek and take alcohol, loss of control in limiting intake, and emergence of a negative emotional state when access to alcohol is prevented. It has been proposed that in alcoholism, the brain reward and stress circuitry fails to maintain homeostatic regulation in the face of chronic excessive drinking and withdrawal but instead develops a set of neuroadaptations to cope with high levels of alcohol exposure [26-28]. Using this conceptual framework, it has been hypothesized that alcoholics continue to drink or relapse to drinking in an attempt to reverse the emotional consequences of the functional dysregulations of their reward and stress systems [13,26-28]. Because of the complexity of alcohol’s effects, a systems biology approach should be particularly well suited to investigate the gene dysregulations associated with alcohol dependence [1]. Here, we used a strategy combining both computational and experimental approaches to identify genes that control the transcriptional regulatory networks that are dysregulated in rats with a history of alcohol dependence.

The present systems biology approach is aimed at identifying key genes that are causally related to the phenotype of interest, rather than on the identification of those that are merely statistically associated with it (that is, gene activity vs. gene expression). This is predicated on the reconstruction (reverse-engineering) of accurate transcriptional regulatory networks (interactomes) for a biologically relevant system, where networks are generated in an unbiased fashion from high-throughput experimental data. This strategy does not lead to the compilation of long lists of differentially expressed genes, but it identifies a much smaller number of key regulators that directly control the differentially expressed genes and are thus much more likely to be causal. Such an approach thus provides high-probability hypotheses for the elucidation of mechanistic and context-specific regulatory events determining the phenotype. Importantly, regulators are not prioritized by their differential expression, which is a poor predictor of regulatory activity, but rather by the differential expression of their targets in a phenotype of interest (for example, history of alcohol dependence). The advantage of using the TF’s differential activity instead of its differential expression is exemplified by Nr3c1/GR, which only showed slight increases in expression in regions where it exhibited the highest levels of activity. In fact, Nr3c1/GR is regulated through multiple transcriptional, post-transcriptional, and post-translational mechanisms [29]. Thus, as expected, the Nr3c1/GR differential activity - as determined by the differential expression of its set of target genes in the regulon - was considerably greater than Nr3c1/GR differential expression. For example, Nr3c1/GR was the most differentially active regulator by MARINa analysis in the core region of the NAc (Figure 2) but only the 544th most differentially expressed gene in the same region (t-statistic: 2.08 and *P* = 0.08). A limitation of the present systems biology strategy is that TFs with few targets are not efficiently identified by MARINa as MRs. However, as true MRs drive the phenotype, they typically have numerous targets. An additional limitation is that a small number of regulators whose regulon significantly overlaps with that of a true MR may result in false positives, although this is mitigated by our pleiotropy analysis. Lastly, ARACNe may invert the predicted direction of regulation when feedback loops are present (the protein negatively regulates its own expression).

We focused our attention on the Nr3c1/GR gene regulatory network because it was found to be one of the highest-ranking master regulators showing differential activation in multiple brain regions of rats with a history of dependence. Additionally, evidence suggests that sustained activation of the hypothalamic-pituitary-adrenal (HPA) axis by alcohol intoxication and withdrawal and consequent overactivation of GRs induce neuroadaptive changes that drive compulsive alcohol drinking in alcohol dependence [13]. In the present study, transcriptional network analysis predicted Nr3c1/GR to be a high-ranking master regulator differentially regulated in multiple brain regions of animals with a history of alcohol exposure including the mPFC, CeA, NAc core and shell, as well as the VTA. We behaviorally validated inhibition of Nr3c1/GR in both the NAc and VTA with the GR antagonist mifepristone (RU38486), which selectively decreased escalated alcohol self-administration during protracted abstinence in rats with a history of dependence. Consistent with these findings, evidence suggests that these brain regions are sensitive to the effects of stress and glucocorticoids. In fact, corticosterone, the main glucocorticoid hormone in rodents (equivalent to cortisol in humans), modulates VTA dopamine cell activity and NAc dopaminergic responses [30-32]. Glucocorticoids have also been shown to act on the NAc to stimulate alcohol consumption in rats [33]. Thus, in light of the role of glucocorticoids in the development of compulsive alcohol drinking in alcohol dependence [13], the present results suggest that glucocorticoid-induced neuroadaptations in the VTA and NAc are key to the neuroadaptations that dysregulate the reward system and contribute to driving excessive alcohol drinking associated with a history of alcohol dependence. While the mesocorticolimbic system, which has origin in the VTA, has an established role in mediating motivation driven by positive reinforcement, VTA circuits are also increasingly implicated in the neurobiological mechanisms behind negative affect, aversion, and dependence [34-37]. The present results point to a role for glucocorticoid-mediated neuroadaptive changes in the VTA and NAc in motivation for alcohol in dependent animals. Additionally, because the VTA and NAc are extensively interconnected with regions of the extended amygdala such as the CeA [38], BLA [39], and BNST [40,41], the glucocorticoid-induced neuroadaptations within these regions are predicted to broadly affect the reward-stress-motivational circuits during excessive drinking associated with ongoing dependence.

Lastly, a measure of intra-network functional similarity was used to generate new hypotheses on transcription co-factors (Tfco) and signaling molecules associated with protracted abstinence in dependent rats. Among signaling molecules showing the greatest degree of co-regulation with GR across the experimental perturbations of the study were Mapk3 (ERK1) [42], previously shown to be regulated by alcohol [42,43], and Med14 (Mediator of RNA polymerase II transcription subunit 14), an ERK-regulated transcriptional regulator [44]; as well as several key regulators of GR such as Smarcad1, Hopx, Nfkb1, Smarca4, Ncor2. These results suggest candidate molecular targets that may be manipulated to affect GR activity, as previously shown in cancer, or for other key MRs driving specific pathologic phenotypes [2,45,46].

The overarching hypothesis behind the present study was that understanding the dysregulations in the gene regulatory network that underlie the neuroadaptive changes associated with excessive alcohol drinking will allow for the identification of new and more effective therapeutic targets for alcohol dependence. Here we showed that gene network analysis of the transcriptional network associated with protracted abstinence in rats with a history of alcohol dependence reveal a large set of transcription factor dysregulations. The gene network predictions were validated for Nr3c1/GR and Psip1, a new alcohol-regulated gene. Nr3c1/GR is a gene of key significance to the paradigm of alcohol dependence-induced excessive drinking; however, the results highlighted a previously unrecognized key role of dysregulation of the Nr3c1/GR regulon in the NAc and VTA in excessive drinking during protracted abstinence. Much animal work to date has focused on the acute reinforcing effects of alcohol, binge-like alcohol seeking, and compulsive-like alcohol seeking during acute withdrawal [47,48]. However, very few studies focus on protracted alcohol abstinence during withdrawal although it is a period highly relevant to human relapse when acute signs of alcohol dependence have dissipated [49]. These results provide support for an integral role of the glucocorticoid system in mediating escalated alcohol intake in animals with a history of alcohol dependence via alterations in key regions of reward and stress circuitry.

## Conclusions

Here we used a systems biology approach to produce a genome-wide transcriptional regulatory network - or transcriptional-interactome - from gene expression profiles of brain regions of the central reward and stress pathways in a rat behavioral model of dependence-associated increased drinking. Using a strategy that infers transcription factor activity through the differential regulation of the direct transcriptional targets, we identified both region-specific and common candidate transcriptional MRs governing the transition from moderate (non-dependent) and excessive (dependent) alcohol drinking brought about by a history of dependence. Results indicate that analysis of interactomes is an effective strategy to identify key regulators and to understand complex psychiatric disorders such as alcoholism. The results also suggest that master regulators like the glucocorticoid receptor (Nr3c1) and Psip1 play a role in the motivation for alcohol in chronic dependence and are potential targets for novel medications in the treatment of chronic alcohol abuse.

## Materials and methods

### Chronic alcohol vapor exposure to induce dependence and mifepristone treatment

Adult male Wistar rats were trained to self-administer alcohol (10% w/v) in operant chambers and made dependent on alcohol by chronic, intermittent alcohol vapor exposure as previously reported [13]. Non-dependent rats were not exposed to alcohol vapor. Rats were removed from alcohol vapor and bilaterally implanted with guide cannulae aiming at the NAc and VTA (coordinates: NAc: +1.7 AP, ±1.35 ML, −7.0 DV; VTA: −6.04 AP, ±0.6 ML, −8.4 DV). After 1 week of recovery and stabilization of responding for alcohol, the rats were injected with mifepristone (30 μg/0.3 μL/side) or vehicle (100% DMSO) in a within-subject Latin-Square design 90 min prior to self-administration sessions. The rats received mifepristone and vehicle into the NAc first and then the same animals received injections into the VTA. Behavioral testing occurred during protracted abstinence (1 to 2 months after removal from alcohol vapor).

All procedures involving animals were approved by The Scripps Research Institute’s (TSRI) Institutional Animal Care and Use Committee and were conducted in PHS-Assured, USDA-registered, AAALAC-accredited facilities according to the PHS Policy, Animal Welfare Act and the Guide for the Care and Use of Laboratory Animals.

### Statistical analysis

Behavioral data are expressed as mean and standard error of the mean (SEM). The data were analyzed using analysis of variance (ANOVA) with treatment (0 vs. 30 μg) as the within-subjects factor and group (dependent vs. non-dependent) as the between-subjects factor. The post hoc comparisons were performed using Fisher’s least significant difference (LSD) test. The accepted level of significance for all tests was *P* <0.05.

### Nr3c1/GR binding site analysis

Promoter regions of the ARACNe predicted targets were defined as a 1,000 bp region, 500 bp upstream and downstream from the transcription start site of each gene. The test set comprised 137 ARACNe-predicted targets of Nr3c1/GR, and the control set comprised 1,000 genes that were in the ARACNe network but not targets of Nr3c1/GR. The occurrence of the Nr3c1/GR binding site was scored using the scoring method outlined in Conlon *et al.* [25] and significance was tested using Fisher’s exact test.

### Gene expression profiles

Gene expression profiles were collected using the Affymetrix Rat Genome 230 2.0 GeneChip® system (31,099 probe sets). Expression measurements were normalized with gcrma (Wu 2004), which adjusts for background intensities in Affymetrix array data, including adjusting for optical noise and non-specific binding. All array files were processed and normalized using R version 2.15.1. All gene expression profiles generated in this study are publically available from GEO at [50] (project accession # GSE60966).

### Cleaner

The normalized gene expression profiles were analyzed using the Cleaner Algorithm [21]. The Cleaner Algorithm performs probe-remapping and probe-correlation analyses for assembly of informative, transcript-specific probe-clusters in Affymetrix expression microarrays. After applying Cleaner, the roughly 31,099 probesets that were originally on the microarray platform were reduced to 7,019 highly-correlated probesets that mapped to 6,715 unique genes. Cleaner R package (version 1.01) was applied to the expression profiles after normalization using R version 2.15.1. The Cleaner-normalized gene expression profile used in our study is available for download from figshare (http://dx.doi.org/10.6084/m9.figshare.1154010).

### Transcription factor classification

To identify transcription factors (TFs), we selected the rat genes annotated as ‘transcription factor activity’ in Gene Ontology and the list of TFs from TRANSFAC [51]. This produced a final list of 898 TFs, from which 664 were present on the Cleaner-mapped expression profile.

### Signaling molecule classification

To identify signaling molecules (Sigs), we selected the rat genes annotated in the GO Biological Process database as: ‘signal transduction’ and in the GO Cellular Component database as GO:0005622 - ‘intracellular’ or GO:0005886 - ‘plasma membrane’. This produced a final list of 2,842 genes, from which 1,100 were present on the Cleaner-mapped expression profile.

### ARACNe

To construct the CNS transcriptional-interactome, ARACNe was applied to the Cleaner-normalized expression profile, as previously described [5]. ARACNe was run with the list of 898 known transcription factors, using adaptive partitioning algorithm, which selects the optimal kernel width for calculating the MI threshold of a specified *P* value. The MI threshold used by ARACNe (MI > = 0.44068) corresponded to the *P* value threshold of 10^−8^ after 100 bootstrap runs. The resulting CNS transcriptional-interactome contained 244,276 statistically significant MIs between the 6,716 genes. The CNS transcriptional-interactome used in this study is available for download from figshare at http://dx.doi.org/10.6084/m9.figshare.1154004. To construct the CNS Signaling Molecule-Interactome, ARACNe was run using the same parameters as the CNS transcriptional-interactome using a list of 1,100 known signaling molecules. The MI threshold used by the adaptive partitioning algorithm (MI ≥0.43795) corresponded to the *P* value threshold of 10^−8^ after 100 bootstrap runs, and the resulting CNS Signaling Molecule-Interactome contained 171,553 statistically significant MIs between 7,018 genes. The CNS Signaling Molecule Interactome used in this study is available for download from figshare at http://dx.doi.org/10.6084/m9.figshare.1154006.

### MARINa and candidate selection

Each TF with more than 25 targets in the CNS transcriptional-interactome was analyzed using MARINa. GSEA was used to assess the enrichment of each TF’s regulon. As a reference, we used a list of genes ranked with the absolute value of the t-statistics obtained by comparing dependent and non-dependent samples. The calculation of the enrichment of each TF produced a list of candidate MRs in each of the sampled regions. MARINa was run with 10,000 probe-shuffling permutations and each TF was given a *P* value based on its normalized enrichment score (NES) as described in Lefebvre *et al.* [9]. Before running MARINa, the CNS transcriptional-interactome was also trimmed at 100 interactions, which meant that for TFs that had greater than 100 ARACNe predicted interactions, only the top 100 most-likely interactions were considered in the calculation of the enrichment score. This was to address a bias in the GSEA, which tends to give a higher score to TFs with larger regulons. Final Master Regulator candidates were defined as TFs with a *P* value <0.01 according to their NES. Full tables of all Master Regulator candidates for all sampled regions and comparisons, along with their associated Normalized Enrichment Scores (NES) and *P* values, can be downloaded from figshare at http://dx.doi.org/10.6084/m9.figshare.1224365.

### Single-sample MARINa and activity correlation analysis

The relative activity of each transcription co-factor (Tfco) and signaling molecule was inferred for each sample using a version of the MARINa algorithm [9] modified to allow signature analysis on a sample-by-sample basis. Single-sample signatures were calculated by differential expression analysis of the sample’s gene expression profile and the average of all gene expression profiles in the same dataset. This analysis allows the inference of the relative activity of each transcriptional regulator or signaling molecule in each sample, based on the relative change in expression of its ARACNe-inferred targets. Specifically, we define the relative activity of a given regulator in a specific sample as the normalized enrichment score (NES) computed by MARINa, based on its gene expression signature. This single-sample MARINa analysis (ssMARINa) was implemented as an R-system package, which is available for download from figshare at http://dx.doi.org/10.6084/m9.figshare.785718.

The correlation of MARINa activity was calculated between each signaling molecule or Tfco that had greater than 25 targets in its regulon, and the significance of the correlation between each pair was calculated by comparing to a null distribution generated by calculating the correlation between random regulons of the same size.

## Additional files

Additional file 1: Tables S1, S2. The number of MR candidates in each region and signature, as well as the overlap between regions. **Table S3.** The MARINa results for Nr3c1/GR in all eight profiled regions, for both alcohol exposed (NaivevsNonDep) and addicted (NonDepvsDep) signatures. **Figure S1.** The results of unsupervised hierarchical clustering of the microarray dataset included in this study. **Figures S2, S3.** The results of hierarchical clustering of MR candidates that were statistically significant after pvalue integration using Stouffer’s method for the alcohol exposed (NaivevsNonDep) and addicted (NonDepvsDep) signatures. **Figures S4, S5.** The top 25 MR candidates for the alcohol exposed and addicted signatures for each region. **Figure S6.** The behavioral effects of Psip1 overexpression in the CeA. **Figure S7, S8.** The results of hierarchical clustering of MARINA-predicted activities for signaling molecules that are also known modulators of Nr3c1/GR activity, as well as TFs and co-TFs that are known modulators of Nr3c1/GR activity. Additional file 2:
**Excel spreadsheet showing ARACNe-predicted targets for the top 25 MR candidates in the alcohol addicted (NonDepvsDep) signature.** Each of the eight profiled regions is shown as a separate sheet in the Excel File and each line in the Excel table corresponds to a single ARACNe-predicted target (represented by a vertical red line in the MARINa plots). The entrez ID and name for the MR candidate and the ARACNe predicted target is shown. Also shown is the rank list metric that was used for the MARINa analysis, which is the absolute value of the t-statistic. The predicted targets for the MR candidates are sorted by decreasing ranked list metric value. Additional file 3:
**Excel spreadsheet showing ARACNe-predicted targets for the top 25 MR candidates in the alcohol exposed (NaivevsNonDep) signature.** Each of the eight profiled regions is shown as a separate sheet in the Excel File and each line in the Excel table corresponds to a single ARACNe-predicted target (represented by a vertical red line in the MARINa plots). Shown are the entrez ID and name for the MR candidate and ARACNe predicted target, as well as the absolute value of the t-statistic for each target. Additional file 4:
**Excel spreadsheet showing ARACNe-predicted targets for the Nr3c1 in the alcohol addicted (NonDepvsDep) signature for the eight profiled regions.** Shown are the entrez ID and name for each ARACNe predicted target, as well as the absolute value of the t-statistic for each target in each region. Additional file 5:
**Excel spreadsheet showing ARACNe-predicted targets for the Nr3c1 in the alcohol exposed (NaivevsNonDep) signature for the eight profiled regions.** Shown are the entrez ID and name for each ARACNe predicted target, as well as the absolute value of the t-statistic for each target in each region. Sweave Documentation: The results of the analysis have also been documented as a Sweave-document, which is designed to outline the set-up and running of MARINa on the Alcohol Addiction dataset. When compiled, the document will run the analysis described in the manuscript and output the full results corresponding to the figures and supplementary figures in the manuscript. The Sweave document, associated pdf document, and files needed for the execution of the Sweave file including the expression file (.exp) and interactome (.rda) can all be downloaded at the following figshare link: http://dx.doi.org/10.6084/m9.figshare.1222965
.
