# Supplementary material for: Identifying candidate drivers of alcohol dependence-induced excessive drinking by assembly and interrogation of brain-specific regulatory networks
Source: Genome Biol. 2015 Feb 2;16(1):68. doi: 10.1186/s13059-015-0593-5 (PMC4410476; doi:10.1186/s13059-015-0593-5)
Supplement: Additional file 1: Tables S1, S2. — The number of MR candidates in each region and signature, as well as the overlap between regions. Table S3. The MARINa results for Nr3c1/GR in all eight profiled regions, for both alcohol exposed (NaivevsNonDep) and addicted (NonDepvsDep) signatures. Figure S1. The results of unsupervised hierarchical clustering of the microarray dataset included in this study. Figures S2, S3. The results of hierarchical clustering of MR candidates that were statistically significant after pvalue integration using Stouffer’s method for the alcohol exposed (NaivevsNonDep) and addicted (NonDepvsDep) signatures. Figures S4, S5. The top 25 MR candidates for the alcohol exposed and addicted signatures for each region. Figure S6. The behavioral effects of Psip1 overexpression in the CeA. Figure S7, S8. The results of hierarchical clustering of MARINA-predicted activities for signaling molecules that are also known modulators of Nr3c1/GR activity, as well as TFs and co-TFs that are known modulators of Nr3c1/GR activity. [file 13059_2015_593_MOESM1_ESM.docx]

**Supplementary Tables**

**Table S1.** Number of Master Regulators (MRs) that were predicted in each region (p < 0.01). The regions are nucleus accumbens core (NAcC), nucleus accumbens shell (NAcSh), ventral tegmental area (VTA), medial prefrontal cortex (mPFC), central amygdala (CeA), basolateral amygdala (BLA), dorsal bed nucleus of the stria terminalis (dlBNST), and ventral BNST (vBNST). **Naive**: alcohol naive control rats; **NonDep**: nondependent alcohol self-administering rats; **Dep**: dependent alcohol self-administering rats.

| **Comparison** | **Region** | **NAcC** | **NAcSh** | **VTA** | **mPFC** | **CeA** | **BLA** | **dlBNST** | **vBNST** |
| --- | --- | --- | --- | --- | --- | --- | --- | --- | --- |
| Naive *vs.* NonDep | MRs | **28** | **56** | **51** | **148** | **42** | **15** | **44** | **51** |
| NonDep *vs.* Dep | MRs | **17** | **30** | **56** | **14** | **28** | **39** | **48** | **15** |

**Table S2.** Overlap of Master Regulators associated with the transition from alcohol naive to nondependent, or nondependent to dependent drinking between different regions. The table shows the p-values associated with the overlap between different regions by Fisher’s exact test and the number of overlapping Master Regulators (underneath). A) shows the results for Naive vs NonDependent and B) shows the results for NonDependent vs Dep rats. Regions that had a significant overlap (p < 0.01) are highlighted in bold.

**Table S2a, Naive vs. NonDependent**

|  | **NAcC** | **NAcSh** | **VTA** | **mPFC** | **CeA** | **BLA** | **dlBNST** | **vBNST** |
| --- | --- | --- | --- | --- | --- | --- | --- | --- |
| NAcC |  | 0.84 | **0.00** | 0.50 | 0.38 | 0.08 | 0.76 | 1.00 |
|  |  | (1) | **(9)** | (5) | (2) | (2) | (1) | (0) |
| NAcSh |  |  | 1.00 | 1.00 | 0.11 | 1.00 | 0.95 | **0.00** |
|  |  |  | (0) | (2) | (5) | (0) | (1) | **(10)** |
| VTA |  |  |  | 0.12 | 0.21 | 1.00 | 0.73 | 1.00 |
|  |  |  |  | (12) | (4) | (0) | (2) | (0) |
| mPFC |  |  |  |  | 0.98 | 1.00 | **0.01** | 1.00 |
|  |  |  |  |  | (3) | (0) | **(14)** | (1) |
| CeA |  |  |  |  |  | 0.03 | 1.00 | 0.70 |
|  |  |  |  |  |  | (3) | (0) | (2) |
| BLA |  |  |  |  |  |  | 1.00 | 0.59 |
|  |  |  |  |  |  |  | (0) | (1) |
| dlBNST |  |  |  |  |  |  |  | 0.93 |
|  |  |  |  |  |  |  |  | (1) |
| vBNST |  |  |  |  |  |  |  |  |

**Table S2b, NonDependent** **vs. Dep**

|  | **NAcC** | **NAcSh** | **VTA** | **mPFC** | **CeA** | **BLA** | **dlBNST** | **vBNST** |
| --- | --- | --- | --- | --- | --- | --- | --- | --- |
| NAcC |  | **0.00** | 0.02 | 1.00 | 1.00 | 0.53 | 1.00 | 1.00 |
|  |  | **(6)** | (4) | (0) | (0) | (1) | (0) | (0) |
| NAcSh |  |  | **0.00** | 1.00 | 0.62 | 0.74 | 1.00 | 1.00 |
|  |  |  | **(8)** | (0) | (1) | (1) | (0) | (0) |
| VTA |  |  |  | 0.21 | **0.01** | 0.43 | **0.01** | 1.00 |
|  |  |  |  | (2) | **(6)** | (3) | **(8)** | (0) |
| mPFC |  |  |  |  | 1.00 | 0.47 | 0.54 | 1.00 |
|  |  |  |  |  | (0) | (1) | (1) | (0) |
| CeA |  |  |  |  |  | 0.72 | **0.00** | 0.08 |
|  |  |  |  |  |  | (1) | **(13)** | (2) |
| BLA |  |  |  |  |  |  | **0.01** | 1.00 |
|  |  |  |  |  |  |  | **(6)** | (0) |
| dlBNST |  |  |  |  |  |  |  | 0.19 |
|  |  |  |  |  |  |  |  | (2) |
| vBNST |  |  |  |  |  |  |  |  |

**Table S3.** MARINa results for Nr3c1/GR in all 8 profiled regions, for both alcohol exposed (Naive vs NonDep) and addicted (NonDep vs Dep) signatures. For each region and comparison the table shows the Normalized Enrichment Score (NES), a measure of TF activity given by MARINa, as well as the associated p-value. The table also shows the Activity Rank of Nr3c1/GR, measured by NES, and relative to all 898 TFs in the interactome, as well as the rank of differential expression (DE Rank). Supplementary Table 3a shows the results of the Naive vs NonDep signature, and Supplementary Table 3b shows the results of the Nondep vs Dep signature.

| **Table S3a** | | | | | |
| --- | --- | --- | --- | --- | --- |
| **Region** | **Comparison** | **NES** | **Pvalue** | **Activity Rank** | **DE Rank** |
| CeA | Naive vs NonDep | 3.014333 | 0.002575 | 19 | 905 |
| NAcC | Naive vs NonDep | 2.551565 | 0.010724 | 31 | 1030 |
| MPF | Naive vs NonDep | 2.464279 | 0.013729 | 164 | 2089 |
| VTA | Naive vs NonDep | 2.113606 | 0.034549 | 95 | 3198 |
| BLA | Naive vs NonDep | 0.569967 | 0.5687 | 421 | 3050 |
| NAcSh | Naive vs NonDep | 0.212906 | 0.8314 | 698 | 6816 |
| vBNST | Naive vs NonDep | 0.121494 | 0.9033 | 776 | 6833 |
| dlBNST | Naive vs NonDep | 0.065972 | 0.9474 | 796 | 5329 |

| **Table S3b** | | | | | |
| --- | --- | --- | --- | --- | --- |
| **Region** | **Comparison** | **NES** | **Pvalue** | **Activity Rank** | **DE Rank** |
| NAcSh | NonDep vs Dep | 4.648268 | 3.35E-06 | 5 | 394 |
| NAcC | NonDep vs Dep | 3.491568 | 0.00048 | 1 | 544 |
| VTA | NonDep vs Dep | 3.423246 | 0.000619 | 19 | 4087 |
| BLA | NonDep vs Dep | 0.598959 | 0.5492 | 497 | 2778 |
| CeA | NonDep vs Dep | 0.56761 | 0.5703 | 467 | 2281 |
| MPF | NonDep vs Dep | 0.365819 | 0.7145 | 590 | 2035 |
| vBNST | NonDep vs Dep | 0.127936 | 0.8982 | 792 | 5964 |
| dlBNST | NonDep vs Dep | 0.041998 | 0.9665 | 812 | 5877 |

**Supplementary Figures**

mPFC


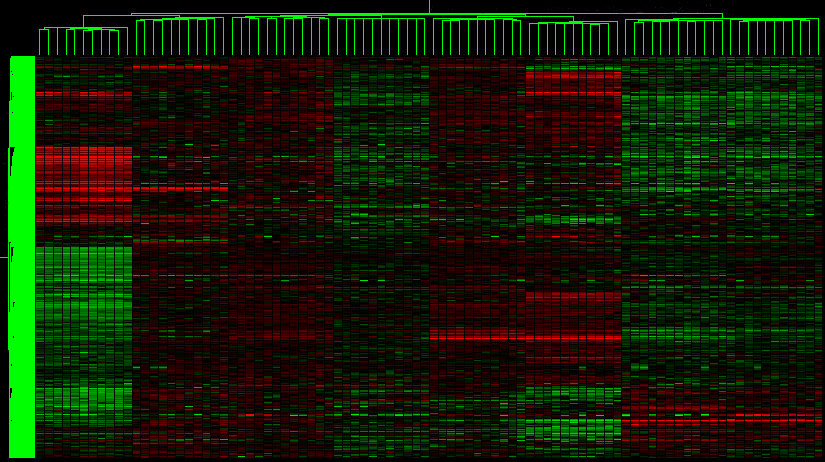


NAcC

NAcSh

CeA

BLA

dlBNST

vBNST

VTA

**Figure S1.** Unsupervised hierarchical clustering of the microarray dataset used for the study. Similar transcriptional programs – as represented by their closeness in the cluster - were displayed by Nac shell (NAcSh) and core (NAcC) sub-regions of the nucleus accumbens and by the extended amygdala regions CeA (central nucleus of the amygdala) and dorsolateral and ventral BNST (dlBNST and vBNST, respectively). Also expectedly, the medial prefrontal cortex (mPFC), a cortical region, clustered with the cortical-like basolateral nucleus of the amygdala (BLA).

**Fig. S2.** Results of hierarchical clustering of MR candidates that were statistically significant after p-value integration using Stouffer’s method for the Naive vs Nondep signature. The colors represent the levels of inferred activities for the MRs, with the most intensely colored MRs being the most active.

**Figure S3.** Results of hierarchical clustering of MR candidates that were statistically significant after p-value integration using Stouffer’s method for the Nondependent vs Dependent signature. The colors represent the levels of inferred activities for the MRs, with the most intensely colored MRs being the most active.

**Figure S4 a-h.**  MARINa results for all regions in the Naive vs. NonDependent signature. The top-25 MR candidates are shown. The regions shown are a) Core of the Nucleus Accumbens (NAcC), b) Shcell of the Nucleus Accumbens (NAcSh), c) Medial Prefrontal Cortex (mPFC), d) Central Amygdala (CeA), e) Basolateral Amygdala (BLA), f) BNST Ventral (vBNST), g) BNST Dorsal (dlBNST), h) Ventral Tegmental Area (VTA). As with figure 2 in the main text, each row of the plot shows the result of MARINa for each MR candidate. The p-value associated with each MR candidate is shown on the left, and the differential expression (Exp) and the MARINA-predicted differential activity (Act) for each MR are shown on the right in shades of red. The number on the right side of the plot indicates the rank of differential expression (Exp) for each MR candidate. The red lines in the middle of the plot indicate ARACNe predicted targets of each MR candidate. The position of each line is determined by its differential expression, with more differentially expressed genes being shown toward the left, and the less differentially expressed genes displayed toward the right. . The identity and differential expression of the ARACNe-predicted targets of each MR candidate (represented by red lines), can be found in supplementary Additional file 3.

**Figure S4a:** Top-25 results of MARINa for core of the nucleus accumbens on the Naive vs. NonDependent signature**.**

**Figure S4b:** Top-25 results of MARINa for shell of the nucleus accumbens on the Naive vs. NonDependent signature**.**

**Figure S4c:** Top-25 results of MARINa for Medial Prefrontal Cortex on the Naive vs. NonDependent signature**.**

**Figure S4d:** Top-25 results of MARINa for Central Amygdala on the Naive vs. NonDependent signature**.**

**Figure S4e:** Top-25 results of MARINa for Basolateral Amygdala on the Naive vs. NonDependent signature**.**

**Figure S4f:** Top-25 results of MARINa for BNST Dorsal on the Naive vs. NonDependent signature**.**

**Figure S4g:** Top-25 results of MARINa for BNST Ventral on the Naive vs. NonDependent signature**.**

**Figure S4h:** Top-25 results of MARINa for Ventral Tegmental Area on the Naive vs. NonDependent signature**.**

**Figure S5 a-g.**  MARINa results for all regions for the NonDependent vs. Dependent signature. The top-25 MR candidates are shown. The regions shown are a) Core of the nucleus accumbens (NAcC), b) Medial Prefrontal Cortex (mPFC), c) Central Amygdala (CeA), d) Basolateral Amygdala (BLA), e) BNST Ventral (vBNST), f) BNST Dorsal (dlBNST), g) Ventral Tegmental Area (VTA). Results of MARINa for core of the nucleus accumbens (NacC) are shown in Fig. 2 in the main text. The identity and differential expression of the ARACNe-predicted targets of each MR candidate (represented by red lines), can be found in Additional file 2.

**Figure S5a:** Top-25 results of MARINa for shell of the nucleus accumbens on the NonDependent vs. Dependent signature**.**

**Figure S5b:** Top-25 results of MARINa for Medial Prefrontal Cortex on the NonDependent vs. Dependent signature**.**

**Figure S5c:** Top-25 results of MARINa for Central Amygdala on the NonDependent vs. Dependent signature**.**

**Figure S5d:** Top-25 results of MARINa for Basolateral Amygdala on the NonDependent vs. Dependent signature**.**

**Figure S5e:** Top-25 results of MARINa for BNST Ventral on the NonDependent vs. Dependent signature**.**

**Figure S5f:** Top-25 results of MARINa for BNST Dorsal on the NonDependent vs. Dependent signature.

**Figure S5g:** Top-25 results of MARINa for Ventral Tegmental Area on the NonDependent vs. Dependent signature**.**


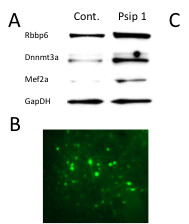


**
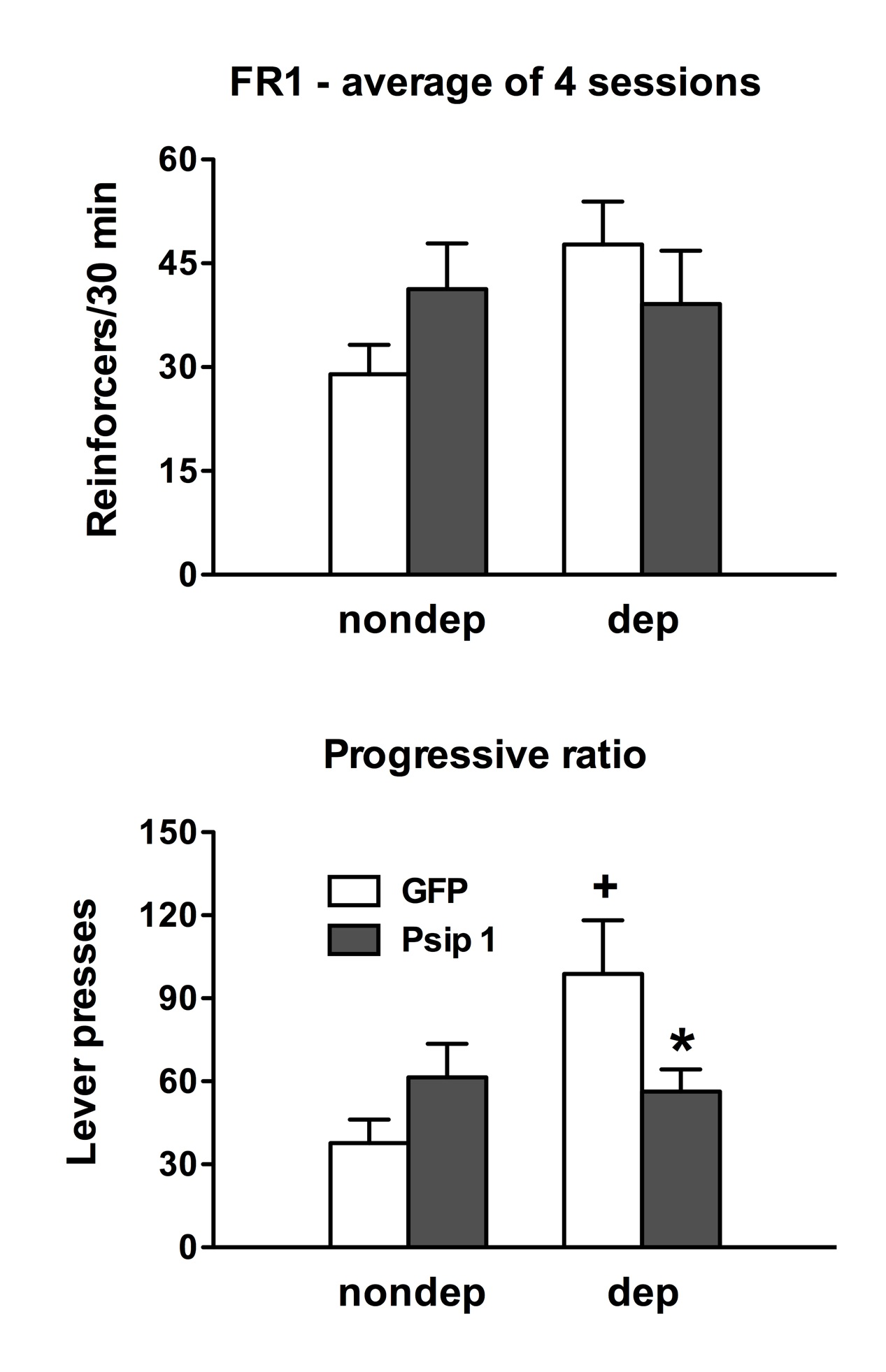
**
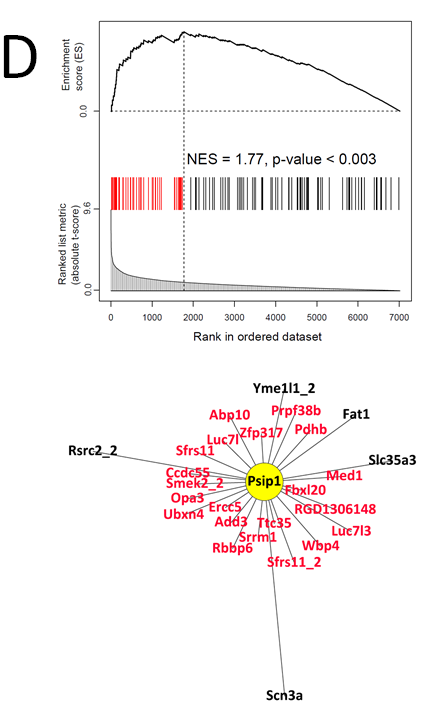


**Figure S6.** The transcription co-activator Psip1 has a functional role in compulsive-like alcohol drinking in alcohol dependent rats. **A)** Validation of Psip1 regulon predicted by the CNS interactome. As indicated by analysis of the CNS interactome, the expressions levels of 3 of Psip1 predicted targets (Rabbp6, Dnmt3a and Mef2a) were increased after transfection of Psip1 in the M213cell line (GapDH was used as a loading control). **B)** Psip1 was transduced in the CeA of Dep and Nondep alcohol self-administering rats by AAV. A control AAV-GFP injection is shown. **C)** Psip1 delivery in the CeA resulted in decreased compulsive-like alcohol drinking in rats with a history of alcohol dependence, but not in nondep rats, as shown by a significant (p < 0.05) reduction in cumulative lever press for alcohol in a progressive ratio schedule of reinforcement, in which the workload for the next reinforcement increases progressively. **D)** Top: targets of Psip1 in the CeA, are differentially regulated in the nondependent *vs.* dependent signature by GSEA. Bottom: Psip1 regulon. Genes in the leading edge of the GSEA analysis are shown in red. These are the genes that contribute most significantly to the enrichment of Psip1, and are the most strongly regulated targets. In the regulon plot, the distance between Psip1 and each target represents the degree of differential expression in the signature. More differentially expressed genes are shown closer to Psip1, and less differentially expressed genes are shown further away. As with the GSEA plot, the genes in the leading edge are shown in red.

**Figure S7.** Results of hierarchical clustering of MARINa-predicted activities for signaling molecules that are also known modulators of Nr3c1/GR activity. Regions/conditions where the genes were found to have increased activity are shown in shades of red. The genes shown were found to have significant correlation with Nr3c1/GR activity across all the regions with a p-value < 0.05.

**Figure S8.** Results of hierarchical clustering of MARINa-predicted activities for TFs and co-TFs that are also known modulators of Nr3c1/GR activity. Regions/conditions where the genes were found to have increased activity are shown in shades of red. The genes shown were found to have significant correlation with Nr3c1/GR activity across all the regions with a p-value < 0.05.
